# The RdRp genotyping of SARS-CoV-2 isolated from patients with different clinical spectrum of COVID-19

**DOI:** 10.1186/s12879-024-09146-x

**Published:** 2024-03-04

**Authors:** Michał Majchrzak, Łukasz Madej, Małgorzata Łysek-Gładysińska, Dorota Zarębska-Michaluk, Katarzyna Zegadło, Anna Dziuba, Katarzyna Nogal-Nowak, Wioleta Kondziołka, Iwona Sufin, Mieczysława Myszona-Tarnowska, Mateusz Jaśkowski, Mateusz Kędzierski, Jadwiga Maciukajć, Jarosław Matykiewicz, Stanisław Głuszek, Wioletta Adamus-Białek

**Affiliations:** 1 Institute of Medical Sciences, Jan Kochanowski University, Kielce, Poland; 2 Institute of Biology, Jan Kochanowski University, Kielce, Poland; 3 CDC Poland Sp. z o.o, Kielce, Poland; 4 Scientific and Research Center ARTMEDIK Sp. z o.o, Kielce, Poland; 5 Meduniv Sp. z o.o, Kielce, Poland; 6 Institute of Health Sciences, Jan Kochanowski University, Kielce, Poland; 7 Specialist Hospital of St. Łukasz, Końskie, Poland; 8 Poviat Healthcare Center, Starachowice, Poland

**Keywords:** *RdRp*, SARS-CoV-2, Severity of COVID-19

## Abstract

**Background:**

The evolution of SARS-CoV-2 has been observed from the very beginning of the fight against COVID-19, some mutations are indicators of potentially dangerous variants of the virus. However, there is no clear association between the genetic variants of SARS-CoV-2 and the severity of COVID-19. We aimed to analyze the genetic variability of *RdRp* in correlation with different courses of COVID-19.

**Results:**

The prospective study included 77 samples of SARS-CoV-2 isolated from outpatients (1st degree of severity) and hospitalized patients (2nd, 3rd and 4th degree of severity). The retrospective analyses included 15,898,266 cases of SARS-CoV-2 genome sequences deposited in the GISAID repository. Single-nucleotide variants were identified based on the four sequenced amplified fragments of SARS-CoV-2. The analysis of the results was performed using appropriate statistical methods, with *p* < 0.05, considered statistically significant. Additionally, logistic regression analysis was performed to predict the strongest determinants of the observed relationships. The number of mutations was positively correlated with the severity of the COVID-19, and older male patients. We detected four mutations that significantly increased the risk of hospitalization of COVID-19 patients (14676C > T, 14697C > T, 15096 T > C, and 15279C > T), while the 15240C > T mutation was common among strains isolated from outpatients. The selected mutations were searched worldwide in the GISAID database, their presence was correlated with the severity of COVID-19.

**Conclusion:**

Identified mutations have the potential to be used to assess the increased risk of hospitalization in COVID-19 positive patients. Experimental studies and extensive epidemiological data are needed to investigate the association between individual mutations and the severity of COVID-19.

**Supplementary Information:**

The online version contains supplementary material available at 10.1186/s12879-024-09146-x.

## Background

In the twenty-first century we struggle with the new coronavirus SARS-CoV-2 causing COVID-19 [1]. This virus, as well as other coronaviruses, uses the enzyme called RNA-dependent RNA polymerase, which determines the number of copies of the virus in the host organism, and this in turn often correlates with the symptoms of the disease [1–4]. General *RdRps* are conserved among viruses [4–6] despite this, numerous mutations in this gene have been identified in SARS-CoV-2 strains [7, 8]. Scientists postulate that the 14408C > T transition is responsible for the increased rate of virus variations [8, 9]. The RdRp enzyme enables the replication of viral RNA to form a replicative form of RNA (a negative-strand RNA particle generated from a template of viral genomic RNA). RNA polymerase creates positive-stranded genomic RNA descendants on the template in replication form. The nature of the coronavirus life cycle also forces the formation of several types of mRNA in the template, which are necessary for the expression of certain viral genes [10–13]. Any changes caused by mutations in the polymerase gene have a great impact on the efficiency of the viral multiplication process, which can be related to its virulence [14, 15]. A typical feature of RdRp polymerase is the lack of a proofreading exonuclease domain. Although SARS-CoV-2 encodes the proofreading exoribonuclease ExoN, it still does not completely eliminate numerous errors during replication (104–106 substitutions per replication) [16, 17]. It is interesting how genomic regions associated with viral replication will evolve and what affects this process? Evolution shows that many viruses that mutate become milder, less recognized by the host, and thus can survive in the environment longer. However, not all of them follow this path, remaining dangerous to the life of their host.

After 3 years, the world has slowly recovered from the COVID-19 pandemic, but there is still a need to study the pathogenicity mechanisms of the virus and the conditions that influence the course of the disease [18–22]. Currently, in most patients, the infection is asymptomatic or mildly symptomatic. Some infected individuals experience a set of flu-like symptoms. The lowest percentage is the subject to the most severe course of the disease, with the risk of severe respiratory disorders and death [23–26]. Most often, these are patients with chronic diseases and impaired immune system, but there are cases of serious diseases without clear explained reasons [27–29]. Our hypothesis is that *RdRp* variability may be the reason for these COVID-19 clinical pictures. Our results appear to have significant application potential in screening for SARS-CoV-2 strains, which poses a high risk of hospitalization for infected patients. The objective of this project was to analyze the SARS-CoV-2 *RdRp* gene quantitatively and qualitatively by Sanger sequencing in patients with different COVID-19 courses. Afterward, the occurrence of the selected mutations was globally verified using GISAID database. Advanced statistical tools were used to observe the correlation between the different COVID-19 courses and the number of mutations, as well as specific mutations.

## Materials and methods

### Study design

Patients from several healthcare facilities in the Świętokrzyskie region of Poland were included in the study: a hospital with a COVID-19 ward in Starachowice, Końskie, Opatów and an outpatient clinic in Kielce (Meduniv Sp. z o.o.). In total, 127 nasopharyngeal swabs were collected between August 2020 and January 2022. Finally, due to technical obstacles at various stages (eg, lack of RNA isolates and PCR products, low quality, or lack of sequencing results), 77 samples were included in the analysis. Patients with COVID-19 were classified according to the clinical signs of SARS-CoV-2 infection and disease severity according to the COVID-19 guidelines [https://www.covid19treatmentguidelines.nih.gov/overview/clinical-spectrum/]: 1st – asymptomatic or presymptomatic infection; 2nd – mild disease; 3rd – moderate disease; 4th – severe disease. The last category of patients (5th – critical illness) was excluded from our study due to the inability to give informed consent to participate in the study. The details of the study group are given in Table 1.

**Table 1:** Cohort characteristics with different clinical spectrum of COVID-19, SD – standard deviation; Q1, Q3 – Quartiles; n – number

spectrum of COVID-19	Age			Sex	
	Mean (SD)	Median (Q1; Q3)	Range	Male n (%)	Female n (%)
1st (*n* = 33)	40.19 (11.62)	37.00 (32.00; 47.75)	52.00	19 (57)	14 (42)
2nd (*n* = 8)	67.38 (21.21)	73.00 (56.25; 82.25)	67.00	5 (62)	3 (37)
3rd (*n* = 24)	65.73 (17.11)	68.00 (52.50; 80.00)	62.00	12 (50)	12 (50)
4th (*n* = 12)	65.42 (16.32)	67.50 (53.75; 75.00)	54.00	5 (41)	7 (58)
Total (n = 77)	54.81 (19.75)	53.00 (37.00; 72.00)	72.00	41 (53)	36 (46)

The laboratory research stages were as follows: RNA isolation from SARS-CoV-2 of deposited samples, cDNA synthesis, PCR reactions specific for the *RdRp* gene, sequencing of PCR products, analysis of sequences in terms of number and type of mutations. The next stage of the research was the retrospective analysis of the occurrence of selected mutations in the world using the GISAID database. The analysis was carried out using the EpiVoc database (https://www.epicov.org/epi3/cfrontend#15a683) and bioinformatics software. The analysis was based on data filtering principles. 15,898,266 of SARS-CoV-2 genome sequences were analyzed, including 7,826,812 of Europe cohort sequences and 93,279 of Polish cohort sequences. The clinical status of the deposited samples was considered for statistical analyses. The COVID-19 cases in GISAID database were described as: mild, asymptomatic, not hospitalized, severe, deceased, released, hospitalized, alive, live, symptomatic and unknown.

### RNA isolation and cDNA synthesis

Nasopharyngeal swabs from patients with COVID-19 were collected in Hanks’ balanced fluid medium enriched with inactivator and nucleic acid protectants (TK Biotech Sp. z o. o., Warszawa, Poland), immediately placed at approximately 4 °C, and transported to the laboratory. The samples were stored at − 80 °C, and total RNA was isolated using the GeneProof PathogenFree RNA Isolation Kit (Imogena Sp. z o.o., Poznań, Poland) according to the manufacturer’s protocol. Pure RNA samples (50 μL) were frozen at − 80 °C until further use.

cDNA synthesis was performed using reagents from Thermo Fisher Scientific MA, USA). Reagents were added to a sterile nuclease-free tube placed on ice sequentially:11,5 μL RNA sample, 1 μL Random Hexamer Primer, 4 μL 5X Reaction buffer, 0,5 μL RiboLock RNase Inhibitor (40 U/μL), 2 μl dNTP Mix (10 mM each), 1 μL Revert Aid Reverse Transcriptase (200 U/μL) in total volume 20 μL. The reaction solution was gently mixed and briefly vortexed. RT-PCR was performed as follows:25 °C for 10 min, 42 °C for 60 min, and 70 °C for 10 min. After synthesis, the samples were frozen at − 20 °C or used for further analysis.

### PCR

**Fig. 1:**
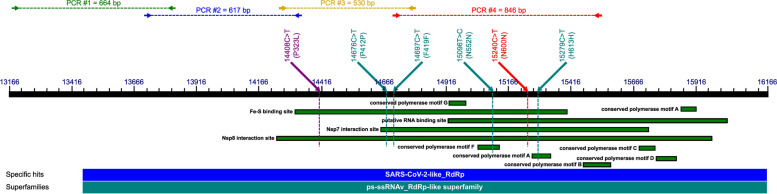
Positions of primer attachment sites and areas corresponding to each of the PCR amplicons, as well as the most specific mutations identified in the *RdRp* gene among the SARS-CoV-2 samples isolated from COVID-19 patients in relation to the sequence encoding the RdRp enzyme in the genome of the SARS-CoV-2 virus. Legend: Mutations shown in blue-green were identified only in viruses collected from the severe illness study group. The mutation shown in red was identified only in viruses collected from the symptomless study group. The mutation shown in purple was identified in both groups. The conserved domains identified by a Conserved Domain Search tool are shown as green boxes underneath the black line representing the sequence. The blue numbers above the black line are nucleotide positions, concordant with the SARS-CoV-2 reference sequence (NC_045512.2). The figure was prepared using (https://inkscape.org/) 1.3 software

cDNA synthesized from the isolated total RNA was used to amplify the N-terminal domain of the *RdRp*-encoding gene of SARS-CoV-2 (ORF1 1a/b). The primers were designed using OligoAnalyzer and OligoCalc online tools, according to the positions of ORF1 a/b polyprotein domains, encoding, among others, four conserved polymerase motifs (A, B, F, and G), forming part of the active site of RNA polymerase enzyme; putative RNA binding domain, responsible for primer binding during RNA replication; Nsp7 and Nsp8 co-factor interaction sites; and the Fe-S co-factor binding site. The positions of each pair of primers, as well as the lengths of the amplified PCR products, primer sequences (oligo.pl), annealing temperature, and their concentrations in the reaction mixture, are shown in Table 2. The amplified areas of N-terminus of the RdRp polymerase are shown in Fig. 1.

**Table 2:** Amplified regions, oligonucleotide primers, amplicons and PCR conditions, Tan – temperature annealing, Pcon – final primer concentration, bp – base pairs. Amplified regions were marked based on SARS-CoV-2 reference strain NC_045512.2 genome

PCR	Amplified region	Primer name	Primer sequence	Amplicon (bp)	T_an_ [°C]	P_con_ [μM]
#1	13,166–13,829	RdRp1F	5′- CACACTGGTACTGGTCAGGC - 3’	664	59	0.3
		RdRp1R	5′- GCATAGACGAGGTCTGCCAT - 3’			
#2	13,712–14,328	RdRp2F	5′- AGGATTGTCCAGCTGTTGCT - 3’	617	57	0.5
		RdRp2R	5′- TGGGTGGTATGTCTGATCCC - 3’			
#3	14,258–14,787	RdRp3F	5′- ATGACTTCACGGAAGAGAGGT - 3’	530	58	0.5
		RdRp3R	5′- AGCAGCATTACCATCCTGAGC - 3’			
#4	14,700–15,546	RdRp4F	5′- TGACTTTGCTGTGTCTAAGGGT - 3’	846	54	0.5
		RdRp4R	5′- GCCGTGACAGCTTGACAAAT - 3’

The concentration of the cDNA samples was measured with DeNovix (DeNovix Inc.) and diluted with Tris-EDTA (Sigma-Aldrich®) solution if necessary. PCR was performed using 12.5 μL DreamTaq Green DNA Polymerase Master Mix (2x) (Thermo Fisher Scientific, MA USA), each primer (synthesized and delivered by oligo.pl) at final concentration specified in Table 2, approximately 20 ng of cDNA and filled with MiliQ water to 25 μl of total volume. DNA amplification was performed using Master Cycler X50 (Eppendorf SE, Hamburg, Germany). The cycling conditions were as follows: denaturation at 95 °C for 3 min, followed by 35 cycles (denaturation at 95 °C for 30 s, annealing (individual temperatures specified in Table 2) for 10 s, and extension at 72 °C for 1 min), followed by a final extension at 72 °C for 10 min. After electrophoresis on 2% agarose gel with the addition of the Syngen Green DNA Gel Stain dye (Syngen Biotech Sp. z o.o., Wrocław, Poland), the PCR products were visualized under UV and subsequently sequenced.

### Sequencing

The sequencing of individual samples consisted of four steps: purification of the PCR products, sequencing reaction, purification of the products resulting from the sequencing reaction, and capillary electrophoresis. Thermo Fisher Scientific (MA, USA) reagent was used for this procedure.

In the first step, PCR products were visually evaluated by agarose gel electrophoresis (band intensity), and the samples were diluted twice if necessary. To 5 μL of the sample solution, 2 μL of ExoSap-IT Express PCR Product Cleanup (cat. 75,001.200.UL) was added. The samples were mixed and centrifuged (5 s at 1000×g). The samples were incubated in a Master Cycler X50 (Eppendorf, SE Hamburg, Germany) for 4 min at 37 °C and 1 min at 80 °C and then placed on ice.

Individual primers (Table 1) were diluted to 3.2 μM. For each primer, a reaction mixture consisting of: 0.5 μL BigDye Terminator v3.1 Ready Reaction Mix (from BigDye Terminator v3.1 Cycle Sequencing Kit, no cat. 4337458), 1 μL BigDye Terminator v1.1 and v3.1 5X Sequencing Buffer (cat. 4,336,697), 6.5 μL deionized water (RNase/DNase-free), 1 μL of primer (3.2 μM), respectively multiplied by the number of samples. The mixture was vortexed and centrifuged (5 s at 1000×g). Nine microliters of the obtained mixture and 1 μL of the PCR product purified in the previous step were then added to the plate. The plate was sealed, vortexed for 3 s, centrifuged (5 s at 1000×g), and incubated in a Master Cycler X50 (Eppendorf, SE, Hamburg, Germany) under the following conditions: denaturation at 96 °C for 1 min, followed by amplification for 25 cycles at 96 °C for 10 s, annealing for 5 s at 50 °C, and extension at 60 °C for 4 min, and 4 °C until the samples were ready for purification.

Then the mixture was prepared per sample: 45 μL SAM solution (no cat. 4376497), and 10 μL XTerminator solution (cat. 4,376,493) vortexed and 55 μL of the solution was added to each well on the plate removed from the thermal cycler to prevent the balls from sinking to the bottom. After resealing with foil, the mixture was vortexed for 20 min at 1800 rpm and centrifuged (2 min at 1000×g).

After changing the foil to septa and checking the buffer level, the plate was inserted into a SeqStudio Genetic Analyzer sequencer (Applied Biosystems by Thermo Fisher Scientific) and sequenced on the Medium Seq_BDX run module. After the process was completed, the results were automatically saved on the disk in ab1 format.

### Data analysis

Chromatograms of the obtained sequences were analyzed using the FinchTV software v.1.4.0. and the sequences were compared with those of the reference strain NC_045512.2, using BLAST. Each nucleotide in the tested sequence was designated as single nucleotide variant (SNV). The amino acid sequence of the RdRp protein was obtained from GenBank (reference sequence: YP_009725307.1). Conserved domain analysis of the amplified areas was performed using NCBI’s Conserved Domain Search tool, available online.

The normality of the data distribution was verified by the Shapiro–Wilk test. Nonparametric unpaired tests (U Mann-Whitney test, ANOVA Kruskal-Wallis test, Fisher-Exact test, two tailed) were used in statistical analyses due to the skewness of the distribution of individual variables (age, sex, number of mutations, and severity of COVID-19), where *p* < 0.05 means statistically significant. Spearman’s correlation was used for variables on an ordinal scale and for skewed variables.

The occurrence of specific SNVs between outpatients and inpatients was determined by OR (odds ratio) and RR (relative risk) with confidence interval (95% CI) and was analyzed statistically using Fisher’s exact, two tailed, *p* < 0.05 was considered statistically significant, zero values were treated as 0.5 [30, 31]. GraphPad Prism, version 9 (San Diego, CA, USA) was used for the analyses and derivation of figures.

The construction of the logistic regression model was preceded by a preliminary selection of predictors through an assessment of their quality using Cramér’s V coefficient. At that stage, a number of predictors were discarded. The remaining predictors were included in the sequential construction of the logistic regression model. To achieve this, the stepwise forward regression was used, and the significance of the difference between the subsequent models that were built was evaluated using an LR (likelihood ratio) test. In the final step, another group of variables was discarded, as they proved to be insignificant. The remaining variables, however, were included in the final version of the model. The predictors’ statistical significance was verified using the Wald test. The goodness of fit was verified using the Hosmer-Lemeshow test (*p* > α; α = 0.05).

## Results

**Fig. 2:**
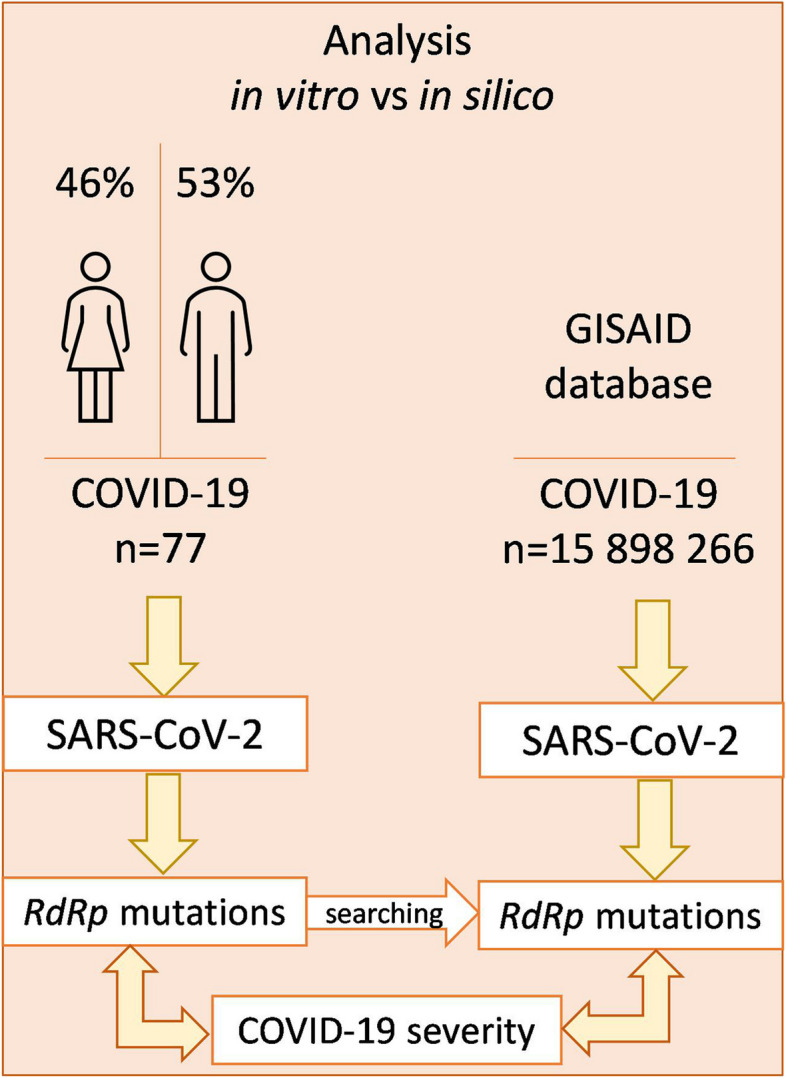
Study design scheme. Legend: n – number of analyzed cases, in vitro – analyses performed in the laboratory including Polish cohort of patients with different COVID-19 course; in silico – analyses performed with the use of the EpiCoV database in the GISAID repository, vs - versus

The studies included prospective (in vitro) and retrospective (in silico) analyses. SARS-CoV-2 samples were collected from patients with various courses of COVID-19 (*n* = 77) and the *RdRp* gene was sequenced. In the first stage, it was verified whether the number of accumulated mutations was correlated with clinical parameters (COVID-19 severity, sex, and patient’s age). Subsequently, we investigated whether there are specific mutations that correlate with the severity of COVID-19 in the studied Polish cohort of patients. The observed correlations were validated at the global level using data from GISAID. The research design scheme is shown in Fig. 2.

### Quantitative mutation analysis

SARS-CoV-2 samples were isolated from patients with different courses of COVID-19, specified in four grades from mild to severe (1st as outpatients and 2nd, 3rd, 4th as inpatients). A total of 77 samples of SARS-CoV-2 were analyzed based on the sequence of four amplified and sequenced regions of the *RdRp* gene: PCR #1 and #2 PCR amplified the N-terminal portion of the *RdRp* coding sequence that did not contain any particular conserved domains (PCR #2 also allowed analysis of the N-terminal portion of the Nsp8 interaction site motif); PCR #3 amplified the sequence corresponding to the N-terminal portions of the Nsp7 and Nsp8 interaction sites, as well as the N-terminal portion of the Fe-S binding site motif; PCR #4 amplified the sequence corresponding to most of the Nsp7 and Nsp8 interaction sites, the Fe-S binding site, as well as conserved polymerase motifs A, B, F, and G and N-terminal portions of putative RNA binding site motifs.

A total of 235 substitutions were detected in the 4 amplified regions of the *RdRp* gene in 77 SARS-CoV-2 samples: 16 (13 different) SNVs in PCR #1 amplicons, 35 (29 different) SNVs in PCR #2 amplicons, 115 (3 different) SNVs in PCR #3 amplicons, and 68 (6 different) SNVs in PCR #4 amplicons. Regions #1 and #2 showed significantly greater genetic variability, but the highest mutation rate was observed in regions #3 and #4 (*p* < 0.0001, ANOVA Kruskal-Wallis test).

**Fig. 3:**
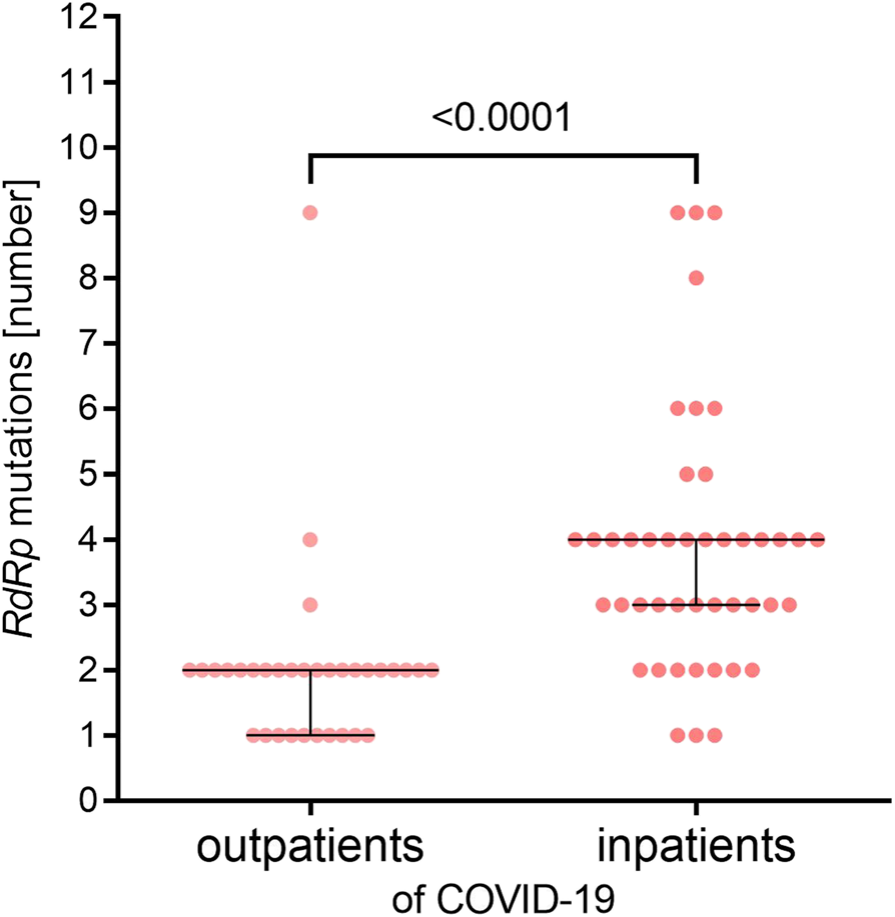
The median number of mutations identified in *RdRp* gene among the SARS-CoV-2 samples isolated from outpatients and inpatients of Polish cohort. Legend: The dots represent participants (*n* = 77) identified with four clinical spectrums of COVID-19: 1st – outpatients, 2nd, 3rd and 4th – inpatients; error bars lines: bottom and top - quartiles Q1, Q3; middle - median; the statistically significant differences (*p* < 0.0001) were measured by U Mann Whitney test, two-tailed

**Fig. 4:**
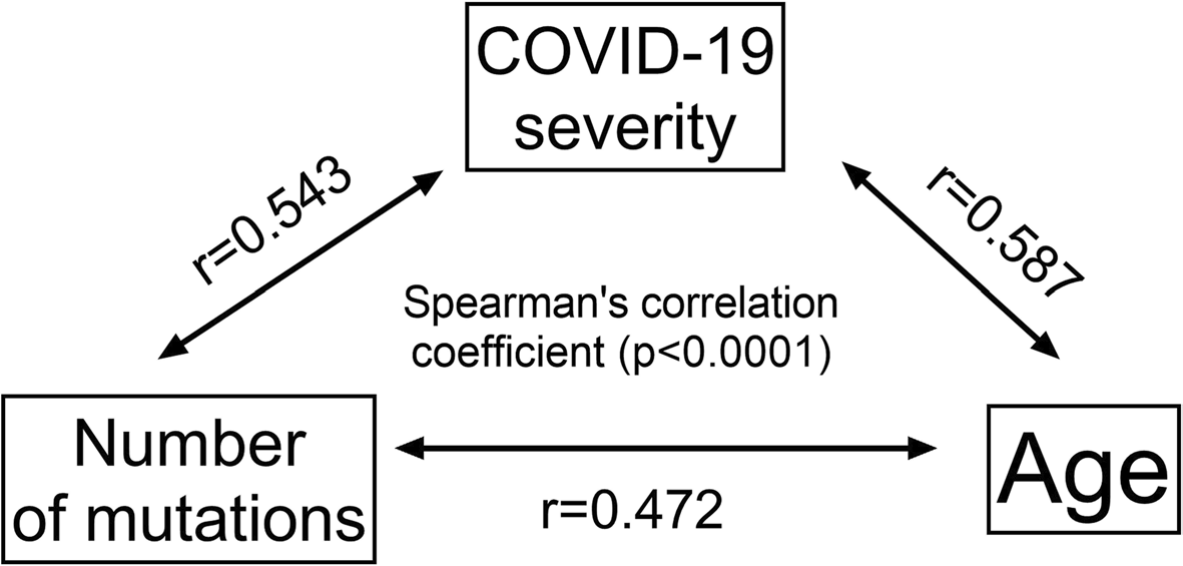
Spearman’s correlation coefficient between the studied variables: number of mutations, COVID-19 severity and age. Legend: COVID-19 severity was identified on a 5-point scale of clinical spectrum (5th degree of COVID-19 was not present in the studied patients), *p* < 0.05 was considered statistically significant, r - Spearman’s correlation coefficient, the arrows indicate the correlation between the variables

**Fig. 5:**
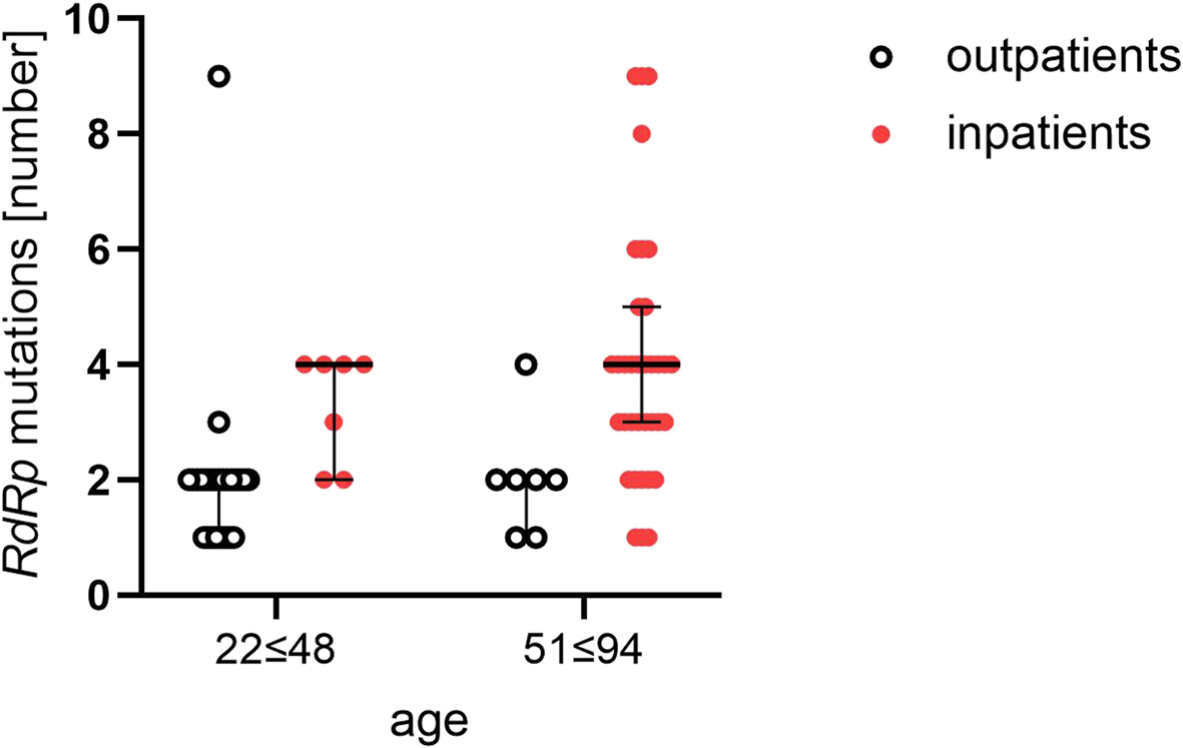
The median number of identified mutations in the *RdRp* gene among the SARS-CoV-2 samples isolated from age-differentiated outpatients and inpatients of the Polish cohort. Legend: The dots represent participants (n = 77) divided into two age groups: 22 ≤ 48 and 51 ≤ 94; outpatients: 1st clinical spectrum of COVID-19; inpatients: 2nd, 3rd and 4th clinical spectrum of COVID-19; error bars lines: bottom and top - quartiles Q1, Q3; middle – median

In total, 51 different SNVs were identified in all 77 tested samples, which were repeated at different frequencies in the tested samples. Patients with the mildest course of COVID-19 were infected with virus strains with the fewest number of mutations (*p* < 0.005, ANOVA Kruskal-Wallis test). There was a tendency towards a positive correlation (increasing number of mutations with a more severe disease course), but perhaps due to the small number of cases, statistically significant differences between groups 2nd, 3rd and 4th could not be demonstrated. Overall, significantly more mutations were detected in the SARS-CoV-2 isolates from outpatients than in those from inpatients (Fig. 3, *p* < 0,0001, test U Mann-Whitney U). Spearman’s correlation analysis was performed for all variables: age, sex, COVID-19 severity (1st – 4th clinical spectrum of COVID-19), and number of mutations. There were statistically significant positive correlations (*p* < 0.0001) between all variables except sex, although a trend toward a higher number of mutations was observed in the female group (data not shown). The strongest Spearman correlation coefficient was observed between the severity of COVID-19 and age (r = 0.587), and a slightly lower coefficient was recorded between COVID-19 severity and the number of mutations (r = 0.543). The weakest correlation coefficient was observed between age and the number of mutations (r = 0.472) (Fig. 4). These correlations are shown in Fig. 5. Patients were divided into younger (22–48 years) and older (aged 51–94 years), and outpatients (1st clinical spectrum of COVID-19) and inpatients (2nd, 3rd and 4th clinical spectrum of COVID-19). Regardless of age, the number of mutations was significantly higher in hospitalized patients (*p* < 0.0001)**.**

### Specific mutation analysis

**Fig. 6:**
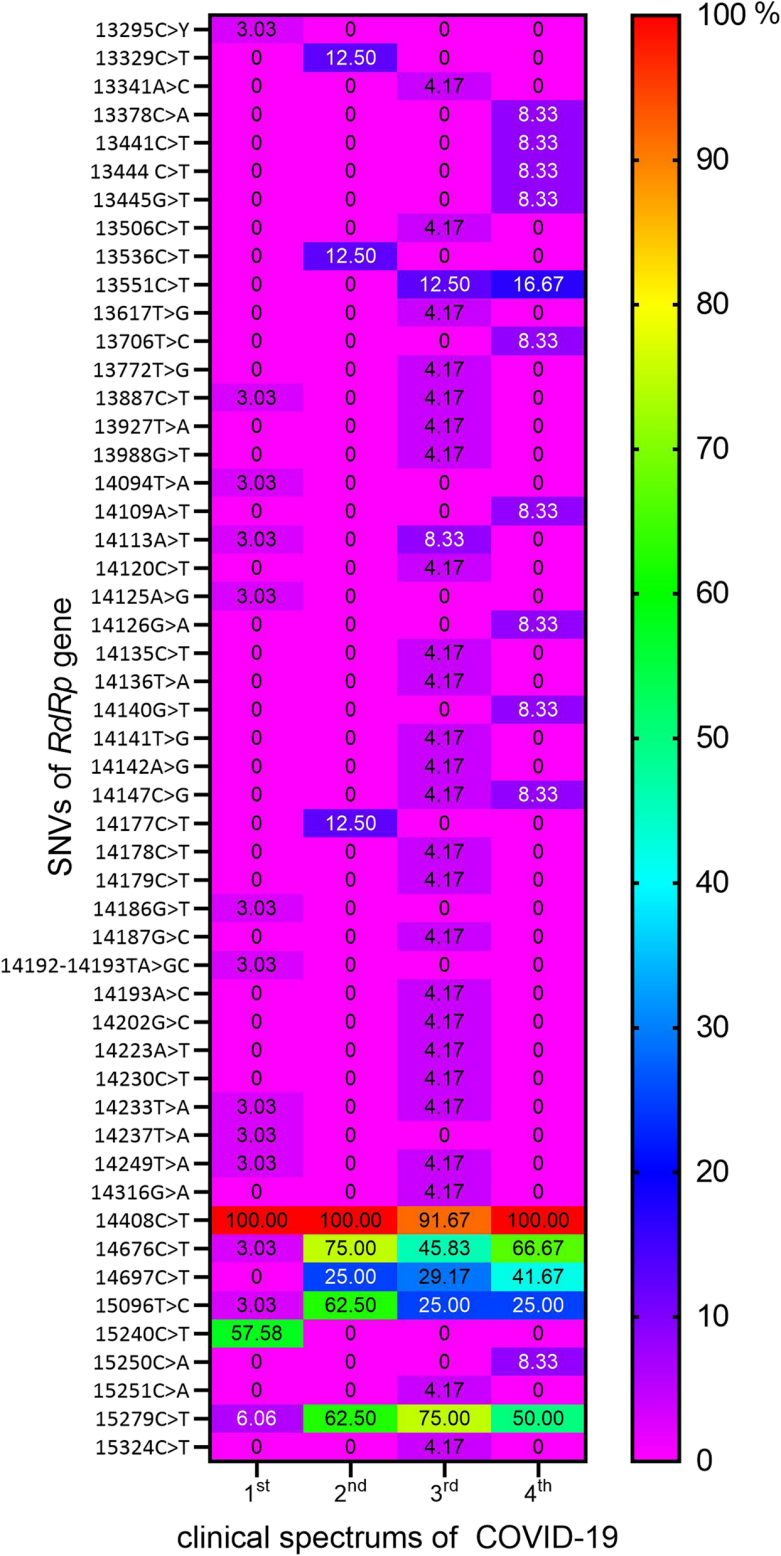
The heat map of the identified single nucleotide variants in the *RdRp* gene among the SARS-CoV-2 samples isolated from patients with different (1st, 2nd, 3rd, 4th) clinical spectrum of COVID-19. Legend: SNVs – single nucleotide variants described by the position in the genome of the SARS-CoV-2 reference strain NC_045512.2 and the type of mutation (nucleotide substitutions marked by >); each cell represents the percentage share of the mutation identified in the clinical group of patients (1st, 2nd, 3rd, 4th), expressed in the appropriate colour

A detailed analysis of the individual mutations allowed us to demonstrate their varied frequencies in the aforementioned groups of patients. The heat map (Fig. 6) reflects the percentage prevalence of each identified SNV (*n* = 51) in SARS-CoV-2 isolated from all patients. As mentioned earlier, the most mutated strains were identified in groups 3 and 4 of the patients. However, 14408C > T (P323L) missense mutation was identified in 97.22% of isolated SARS-CoV-2 samples (only two SARS-CoV-2 isolates did not possess this mutation). Many of the identified mutations were present in only one group of patients (1st, 2nd, 3rd or 4th). Generally, the nucleotide sequences obtained for both inpatients and outpatients showed different patterns of mutations in all sequenced areas of the *RdRp* gene, but only five mutations showed a statistically significant correlation with the clinical groups of patients (Table 3). Among them, one silent mutation (15240C > T; N600N) was present only in outpatients’ group (57.57% of samples), and other 4 silent mutations were significantly more common in groups of inpatients (groups 2nd, 3rd, 4th) compared to outpatients (group 1st). They were 14676C > T (P412P), 14697C > T (F419F), 15,096 T > C (N552N) and 15279C > T (H613H), occurring in mean 62.50%,, 31.94, 37.50 and 62.50% of the samples, respectively (Fig. 6). Both OR and RR indicate many times more frequent occurrences of these SNVs, indicating a high risk of hospitalization in patients infected with such SARS-CoV-2 strains.

**Table 3:** Specific *RdRp* single nucleotide variants of SARS-CoV-2 correlated with the risk of hospitalization of COVID-19 patients

SNV	AA	1st (n = 33)	H (*n* = 44)	1st correlated with H				
				OR	95% CI	RR	95% CI	*p*-value
14676C > T	P412P	1	25	42.10	5.27–336.30	18.75	2.67–131.41	< 0.0001
14697C > T	F419F	0	14	31.85	1.82–557.08	21.91	1.35–354.54	0.0002
15,096 T > C	N552N	1	14	15.93	1.84–120.61	10.50	1.45–75.89	0.0013
15240C > T^a^	N600N	19	0	119.68	6.79–2108.97	51.61	3.22–825.07	< 0.0001
15279C > T	H613H	2	29	29.96	6.29–142.59	10.87	2.79–42.37	< 0.0001

1st – number of asymptomatic COVID-19 patients (outpatients), *H* number of hospitalized (2nd, 3rd and 4th spectrum) COVID-19 patients (inpatients), *n* number of all COVID-19 patients in the group, *OR* odds ratio between groups, *RR* relative risk between groups (1st vs. H), *CI* Confidence interval, *SNV* single nucleotide variant, *AA* Amino acid variant; *p*-value was measured by Fisher’s exact test, two-sided, *p* < 0.05, was considered statistically significant; zero values were treated as 0.5; ^a^ - negative correlation

Furthermore, analysis of the coexistence of these mutations (the presence of at least one of them) showed that only two outpatients were infected with the virus with these mutations (patient no. 68S - SARS-CoV-2 with 15279C > T; patient no. 36S - SARS-CoV-2 with 14676C > T, 15096 T > C, 15279C > T). Instead, 81% of hospitalized patients were infected with SARS-CoV-2 strains with at least one of the mentioned four mutations (8/8 of 2nd group; 18/24 of 3rd group, 10/12 of 4th group). This indicates that the set of four SNVs occurs almost 70 times more often in inpatients and gives a 4.62 times higher risk of hospitalization (RR = 4.62; 95% CI =2.64–8.82; OR = 69.75, 95% CI =13.79–314.6; *p* < 0.0001, two-sided Fisher’s exact test). We did not detect any associations between sex, age, and occurrence of these mutations (data not shown).

### Logistic regression analysis

All studied variables (age, gender, severity of COVID-19, hospitalized, outpatients) in relation to the number of mutations and in relation to the individual mutations were used for regression analysis. We described only those which revealed statistical significance. The evaluation of type of advice (a dependent variable in the logistic regression model) was defined as a dichotomous variable that assumed the following variants: number of mutations, specific mutations, inpatients, outpatients, COVID-19 severity (1st, 2nd, 3rd, 4th), age, gender. We observed five models (A, B, C, D, E) a final form of the logistic regression model with qualitative and quantitative explanatory variables indicated the determinants affecting the analyzed qualitative dependent variables (dichotomic) (Table 4).

**Table 4:** The models of predictors in the variability of *RdRp* gene of SARS-CoV-2 based on the logistic regression analysis

Model	Modeling to	Variable (reference variant)	LR	OR (95% Cl)	*p*-value
A	Female	Constant term	−1.003	0.367 (0.147–0.913)	0.031
		Number of mutations	0.276	1.318 (1.013–1.715)	0.041
B	> 1 mutation	Constant term	−2.832	0.059 (0.015–0.232)	< 0.001
		Spectrum of COVID-19	0.811	0.250 (1.129–4.486)	0.021
C	> 2 mutations	Constant term	−4.302	0.014 (0.002–0.104)	< 0.001
		Spectrum of COVID-19	0.968	2.633 (1.451–4.778)	0.001
		Age	0.037	2.633 (1.002–1.075)	0.041
D	Hospitalized	Constant term	−6.118	0.002 (0.000–0.049)	< 0.001
		15279C > T	3.210	24.788 (3.906–157.302)	< 0.001
		Age	0.105	1.111 (1.050–1.176)	< 0.001
E	> 48 years old	Constant term	−0.938	0.391 (0.181–0.846)	0,017
		15240C > T	−1.259	0.284 (0.090–0.897)	0,032
		15,096 T > C	2.154	8.615 (1.032–71.936)	0.047
		Hospitalized	2.603	13.508 (4.423–41.254)	< 0,001
		Spectrum of COVID-19	1.287	3.623 (2.009–6.534)	< 0,001

The variables - number of mutations, specific mutation, age, gender, spectrum of COVID-19 (1st, 2nd, 3rd, 4th), hospitalized patients, outpatients) were used for the analysis. *LR* Estimate of the logistic regression parameter

Regarding the number of mutations, it was found that women have a statistically significantly higher chance of having more mutations than men (A). It has been shown that the increasing number of mutations increases the risk of a more severe course of COVID-19, especially in older patients (model B, model C). In the model D the chance of occurrence of the 15279C > T mutation was almost 25 times statistically significantly higher in hospitalized patients than in outpatients. Furthermore, the older the patient, the greater the risk of hospitalization. The 15240C > T mutation occurred statistically significantly less frequently in older people (> 46 years) than in younger people (≤46 years), while the 15,096 T > C mutation - on the contrary, occurred statistically significantly more often in older patients (> 46 years). Those patients (> 46 years old) infected with the virus with the mentioned mutations had a higher risk of hospitalization, which also correlated with the clinical spectrum of COVID-19 (model E).

### GISAID analysis

**Fig. 7:**
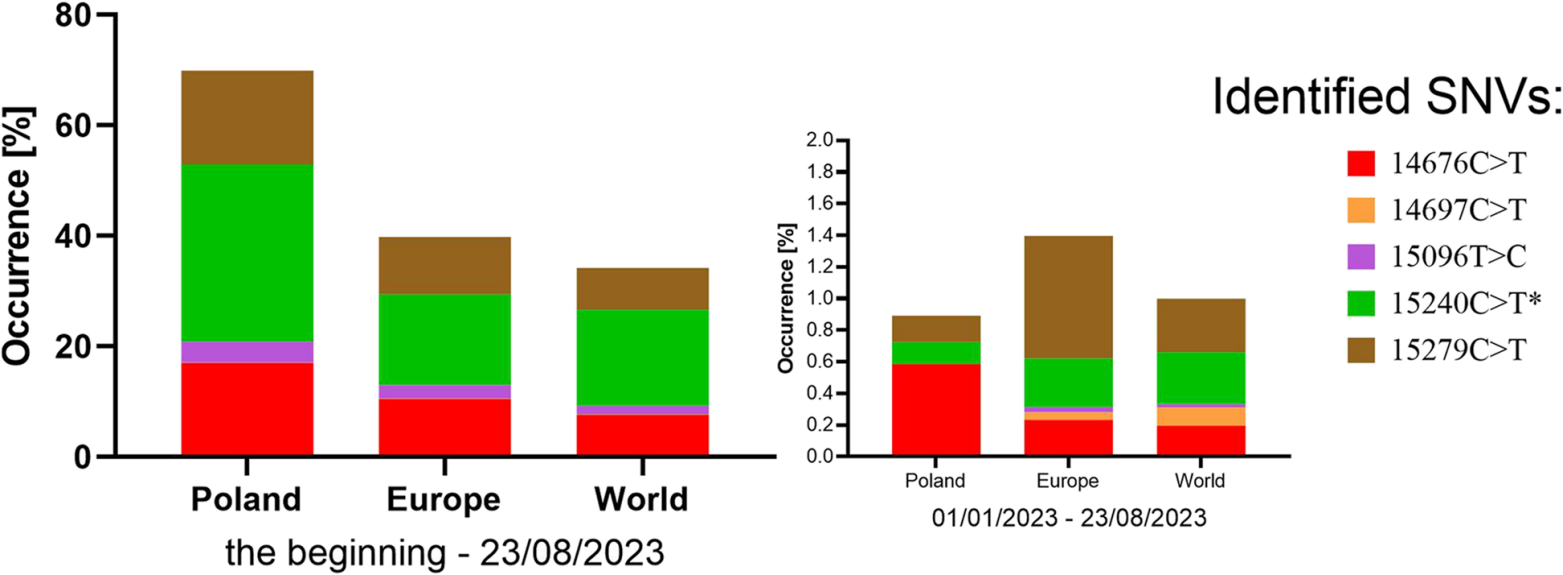
The percentage of occurrence of selected *RdRp* single nucleotide variants in the GISAID repository deposited with SARS-CoV-2 genome sequences. Legend: SNVs – single nucleotide variants described by the position in the genome of the SARS-CoV-2 reference strain NC_045512.2 and the type of mutation (nucleotide substitutions marked by >), *negative correlation between the number of positive samples and COVID-19 severity; Data was individually analyzed for Poland, Europe and the world, in all registered genomic sequences and in data deposited only in 2023. The occurrence of SNVs was analyzed using the EpiCoV database

The occurrence of 14676C > T (P412P), 14697C > T (F419F), 15,096 T > C (N552N), 15240C > T (N600N) and 15279C > T (H613H) was analyzed in silico in genome sequences deposited in the GISAID repository. The number of genome sequences in which individual SNVs were identified, and the number of all genome sequence submissions were presented for Poland, Europe and the world (Table 5). The data applied to all sequences reported so far, and only for the year 2023. The occurrence of SNVs was analyzed using the EpiCoV database. All SNVs were identified in the database but at different frequencies (Fig. 7). The highest prevalence in Poland was 15240C > T (32.09% of all submissions). This SNV was also the most common in the world and Europe but was more typical for Poland; it occurred 2.40 and 2.25 times more often (OR) than in Europe and the world (*p* < 0.0001, Chi-square, two-sided), respectively. Among other SNVs: 14676C > T and 15279C > T had the highest prevalence in Poland (17% of all submissions), as well as in Europe, and worldwide (10 and 7% respectively). Similarly, in Poland 14676C > T occurred 1.75 and 2.47 times more often (OR) than in Europe and the world, respectively (*p* < 0.0001, Chi-square, two-sided). For 15279C > T in Poland it occurred 1.95 and 2.47 times more often (OR) than in Europe and in the world, respectively (*p* < 0.0001, Chi-square, two-sided). The least common variant in Poland, Europe, and the world was 14697C > T. The occurrence of all these SNVs have dropped drastically when analyzing the first half of 2023 (median is 0.16%, range 0.00–0.77%) of all deposited genomes. Two of the rarest (14697C > T, 15096 T > C) have not yet appeared in Poland. The prevalence of the common 15240C > T has clearly decreased, and the 14676C > T is now much more common than the others studied, in Poland. Interestingly, the proportion of 15240C > T occurrence is now much lower in Poland in comparison to Europe and worldwide. 15279C > T is currently the most common among the analyzed SNVs and the proportion of 14697C > T has clearly increased, this is not valid for Poland. The occurrence profile of the studied SNVs differs significantly between Poland, Europe, and the world.

**Table 5:** The occurrence of selected RdRp single-nucleotide variants in SARS-CoV-2 genome sequences deposited in the GISAID repository

SNV	AA	Region and time range					
		The beginning – 23/08/2023			01/01/2023–23/08/2023		
		Poland	Europe	World	Poland	Europe	World
14676C > T	P412P	15,903	819,301	1,218,173	21	716	1778
14697C > T	F419F	27	13,215	18,734	0	153	1033
15,096 T > C	N552N	3553	185,547	240,630	0	97	195
15240C > T	N600N	29,934	1,283,763	2,758,163	5	949	2966
15279C > T	H613H	15,789	816,838	1,209,654	6	2387	3079
All records:		93,279	7,826,812	15,898,266	3590	308,277	905,120

*SNV* Single nucleotide variant, *AA* Amino acid variant; all records: total number of genomic sequences deposited in the GISAID database at a given time and region. The occurrence of SNVs was analyzed using the EpiCoV database

We also differentiated the occurrence of detected SNVs according to the patient’s status available in the GISAID database. Among the deposited SARS-CoV-2 genomes, the following statuses were distinguished: mild, severe, asymptomatic, symptomatic, not hospitalized, hospitalized, released, deceased, alive, live, unknown in Poland, Europe, and the world (Tab. S2). We chose the most analogous statuses to our groups to compare the relationship between the occurrence of individual SNVs and the severity of COVID-19. Asymptomatic patients with COVID-19 were compared to the hospitalized and severe (Table 6). All SNVs confirmed the clinical correlation (significantly increased risk of hospitalization and severe COVID-19), except for 14697C > T. 15240C > T also confirmed this association, contrary to our results (“protective mutation”). We also analyzed the occurrence of these SNVs only in 2023, the correlation was maintained only for 15279C > T (OR = 2.5, 95% CI =1.24–5.13; RR = 1.44, 95% CI =1.10–1.70; *p*-value = 0.011, Chi-square or Fisher’s exact test, two-sided). This mutation among those analyzed is also the most common in the world today. The remaining ones (14676C > T and 15240C > T) showed a similar association, but due to the small number of confirmed samples, the statistical significance was not confirmed.

**Table 6:** Specific *RdRp* single nucleotide variants of SARS-CoV-2 correlated with the severity of COVID-19 according to GISAID database

SNV	AA	A (*n* = 14,938)	H (*n* = 83,979)	A correlated with H				
				OR	95% CI	RR	95% CI	*p*-value
14676C > T	P412P	512 (3.4%)	6998 (8%)	2.56	2.33–2.80	1.12	1.09–1.11	< 0.0001
14697C > T	F419F	10 (0.06%)	35 (0.04%)	1.61	0.77–3.24	1.09	0.97–1.33	0.18
15,096 T > C	N552N	74 (0,49%)	1704 (2%)	4.13	3.27–5.21	1.13	1.12–1.14	< 0.0001
15240C > T	N600N	951 (6.3%)	13,261 (15%)	2.76	2.57–2.95	1.12	1.11–1.12	< 0.0001
15279C > T	H613H	501 (3.35%)	7011 (8.3%)	2.62	2.39–2.87	1.11	1.10–1.12	< 0.0001
SNV	AA	A (n = 14,938)	severe (*n* = 955)	A correlated with severe				
				OR	95% CI	RR	95% CI	*p*-value
14676C > T	P412P	512 (3.4%)	64 (6.7%)	2.02	1.54–2.63	1.9	1.50–2.41	< 0.0001
14697C > T	F419F	10 (0.06%)	0 (0.00%)	–	–	–	–	–
15,096 T > C	N552N	74 (0,49%)	24 (2.51%)	5.2	3.29–8.21	4.17	2.89–5.81	< 0.0001
15240C > T	N600N	951 (6.3%)	79 (8.27%)	1.33	1.05–1.68	1.30	1.04–1.62	0.02
15279C > T	H613H	501 (3.35%)	68 (7.12)	2.21	1.69–2.87	2.06	1.63–2.59	< 0.0001

A number of COVID-19 patients with “asymptomatic” status, H number of COVID-19 patients with “hospitalized” status; severe - number of COVID-19 patients with “severe” status, *n* number of all COVID-19 patients in the group, *OR* odds ratio, *RR* Relative risk, *CI* Confidence interval, *SNV* Single nucleotide variant, *AA* Amino acid variant; *p*-value was measured by Chi-square, two-sided, *p* < 0.05, was considered statistically significant; **−** - not analyzed

## Discussion

Since the first SARS-CoV-2 strain responsible for COVID-19 was identified at the end of 2019, almost 3000 lineages have been reported worldwide to date [32, 33] with the most common Alpha (B.1.1.7 and Q.x), Delta (B.1.617.2 and AY.x), and Omicron (B.1.1.529 and BA.x). These variants differ genetically and exhibit different dissemination and pathogenicity [34–37].

Recent years have shown the natural and rapid evolution of RNA viruses, such as SARS-CoV-2, where it is clearly visible that natural selection promotes the survival of less virulent strains [38]. Lythgoe et al., [39] found also that most mutations in the acute stage of the pandemic were lost, and few mutations were fixed. In our study, we also carried out a molecular analysis of selected regions of the SARS-CoV-2 genome to assess their variability and the obtained results seem to confirm this. In mid-2020 (mostly inpatients) we detected many single nucleotide variants that were rarely seen 6 months later (mostly outpatients), but also some specific SNVs were strongly correlated with the severity of COVID-19 (Table 3, Fig. 6). This is also consistent with the in silico analysis of the GISAID data (Table 5, Fig. 7), where the occurrence of the five selected SNVs dropped drastically in 2023, but it should be emphasized that they are still being detected.

Generally, the number of mutations in the analyzed ORF1ab region showed a strong positive correlation with the severity of COVID-19, especially when comparing outpatients with hospitalized patients (Table 4, Fig. 3) and the increasing age of patients. This statement agrees with the evolution of the SARS-CoV-2 genome [38, 40, 41]. Maurya et al., [41] also observed a higher number of mutations per sample in mortality cases than in convalescents. We also confirmed that younger patients had a milder course of COVID-19, which is now well described [27]. The logistic regression model allowed to determine predictors for the analyzed variables (sex, age, number of mutations) affecting the occurrence of the risk of severity of COVID-19. We can assume that younger females have lower risk of hospitalization due to COVID-19. This may be related to the generally better immune condition in females and younger patients [27, 42–44].

We identified a wide variety of mutations, appearing individually or together with others, all mutations are shown in Fig. 6 but only 6 of them caught our attention, their *loci* are marked in Fig. 1. At first glance, it is obvious that many different mutations appearing in both PCR-covered areas #1 and #2 PCR covered areas do not affect any of these conserved domains. This was true for both mild and severe symptoms in the study groups. In the case of PCR #3-and PCR #4-covered areas, the situation was quite different. The first interesting substitution is 14408C > T (P323L), which overlaps both the Nsp8 interaction site and the Fe-S cofactor binding site. This mutation was described as typical for Europe in 2020 by Pachetti et al., [9] and it has been fixed in all SARS-CoV-2 strains such as Alpha, Beta, Delta, Omikron [45]. This mutation spread quickly, but its frequency varies depending on the region of the world, the lowest in Asia [46, 47]. Toyoshima et al., [48] and Biswas and Mudi [7] reported significant positive correlations of 14408C > T (*RdRp* encoding region) and 23403A > G (S protein encoding region) with death and severe cases of COVID-19. These missense mutations often occur together [46]. This clearly contradicts our observation that 14408C > T was present in almost all the isolates (*n* = 75/77). Does this mean that this mutation is no longer associated with the virulence of SARS-CoV-2 strains, or has it never been? Many mutations may appear simultaneously and spontaneously during the evolution of the virus, but not all of them are responsible for its virulence.

The remaining five mentioned mutations showed a strong correlation with inpatients and outpatients (Table 3). The 14676C > T (P412P) and 14697C > T (F419F) substitutions were many times more common in inpatients than in outpatients, and they overlapped the same domains and the Nsp7 interaction site. However, these two substitutions were not associated with amino acid changes in their corresponding codons. Both substitutions changed the third position of associated codons. This observation was also true for both groups. In the case of PCR #4-covered area, the findings were even more interesting. In the case of the study group for severe symptoms, two substitutions, 15,096 T > C (N552N) and 15279C > T (H613H), overlapped conserved polymerase motifs F and A, respectively, which are crucial from the point of view of viral enzyme activity [49]. These mutations were also significantly more common in hospitalized patients and were replaced by a single substitution 15240C > T (N600N) in 57% of outpatients, which does not overlap any of the polymerase conserved motifs, instead overlapping almost all other conserved domains (Nsp7 / Nsp8 interaction site; Fe-S binding site as well as putative RNA binding site). Xiong et al., [45] describe it as a common mutation in the Omicron. They also identified 14676C > T as a common mutation and 15279C > T as a rare mutation in Alpha. It should be emphasized that the 14697C > T and 15,096 T > C mutations detected in our population have not been described by other authors so far, as well as the other remaining single mutations in the first and second amplified regions of ORF1ab (Fig. 6). These SNVs (14697C > T and 15,096 T > C) were also rare in our study, consistent with the GISAID database analysis (Table 4). Only 15324C > T mutation has been described by other authors as common [7, 8, 50] which is not in line with our observations (only 4.17% of all isolates). Compared, the presence of the above-mentioned five SNVs in our research was consistent with the GISAID data submitted in Poland.

We extended our GISAID analysis to include the patient’s status. Despite the large number of missing data, it was possible to determine the frequency of individual SNVs in the world depending on the severity of COVID-19 (Table 5). It should be noted that some categories seemingly antagonistic (e. g. asymptomatic - symptomatic or not hospitalized - hospitalized) were not good choices for the analysis. They can falsify the correct statistical analysis due to an ambiguous classification of patients. Therefore, we selected the most distinct and extreme status of patients with COVID-19 (asymptomatic versus severe or hospitalized), so that they best match the criteria included in our prospective studies. The GISAID analyses of the data confirmed our finding that SARS-CoV-2 infection with the presence of at least one of tested variants (14676C > T, 15096 T > C, 15279C > T) increases the risk of severe COVID-19 and hospitalisation 2–5 times. Furthermore, the presence of the 15279C > T variant correlating with this risk is still present in 2023. The other two SNVs show similar trend but due to their rarity they did not confirm statistical significance. Due to divergent correlations, we did not include 15240C > T in our final conclusions. This requires further observations, but the application potential is clear. However, we emphasize that regional analyzes are important for epidemiological studies of strains with high virulence potential. We agree with Ugurel et al. [47] the associations between disease severity and the local spread of a given strain may no longer be valid at the global level. This is analogous to the case of bacteria, where local drug resistance profiles serve as guidelines for antibiotic therapy. The monitoring of 14676C > T, 15096 T > C, 15279C > T is epidemiologically justified. Logistic regression models indicated that 15,096 T > C and 15279C > T additionally increase the risk of severe COVID-19 in older patients. Generally, our quantitative and qualitative analysis indicate that mutation rate monitoring of SARS-CoV-2 strains, with particular emphasis on the above-mentioned mutations, may imply an increased risk of hospitalization of infected patients. This may allow for earlier prevention against the spread of these strains among people, increased surveillance of infected patients, and the implementation of a different method of treating these patients. However, it should be emphasized that our results are only a starting point for such conclusions, more extensive epidemiological studies should be conducted. Furthermore, experimental studies could explain how and whether selected mutations are related to strain virulence. The severe acute respiratory evolution caused by SARS-CoV-2 has evolved in recent years. It appears that strains with high virulence were slowly displaced from the population. Although the occurrence of the discussed SNVs has decreased drastically by 2023, they are still present in the SARS-CoV-2 population. This could mean that its prevalence is low but stable, posing a risk of severe COVID-19.

It should also be added that all these substitutions seem to be silent mutations, the nucleotide changes correspond to the third position of each respective codon, and thus they do not cause any amino acid change, being apparently silent mutations. Conserved polymerase motifs A – G seem to be crucial from the point of view of formation of the enzymatically active site [51]; any mutation in these vital areas could theoretically affect the overall enzymatic efficiency of RNA polymerase. For example, these specific mutations may increase viral fitness and so increasing severity of the disease. At the same time, both mutations were absent in outpatient strains isolated, suggesting that selective pressure aimed at the selection of milder disease-causing strains helped remove them from the polymerase gene. The appearance of these silent mutations may also be associated with a codon usage bias, which may occur between the AAT and AAC codons (for 15,096 T > C; N552N) and CAC and CAT (for 15279C > T; H613H). However, the consequence of silent mutations may be a change in the rate of protein biosynthesis. The use of a more frequent codon, including human codons, may have accelerated the translation process and thus viral replication [52–54]. Other reports indicate that mutations in rare codons within highly expressed genes such as *RdRp* can influence the translation of other genes and even change proteome-wide by reducing the availability of the corresponding t-RNAs [55]. However, the possible impact of these mutations on viral polymerase enzymatic activity needs to be verified. The last analysis of SARS-CoV-2 genomes revealed a significant prevalence of C > T mutations (approximately 45% of all mutations) [47, 56], which is close to our observations (37% of all identified mutations). This may be the result of the host’s antiviral activity of APOBEC cytosine deaminases [57, 58]. Kim et al., (2022) proved that APOBEC3 promotes the replication of SARS-CoV-2 [57]. In our study, 19/52 mutations and all five associated with COVID-19 severity were C > T substitutions, confirming the host-dependent evolution of the virus.

We also analyzed the 5 selected mutations in silico using a newly developed tool for genomic analysis of SARS-CoV-2 – named as COV2Var. This publicly available function annotation database of SARS-CoV-2 genetic variation (http://biomedbdc.wchscu.cn/COV2Var/) was developed by Feng et al. [59]. The mutation 14676C > T, 15240C > T and 15279C > T showed the same protein mutation, similar occurrence rate, and correlation compared to our observations. These data also indicate an increased risk of occurrence of mutations depending on age groups and gender in some cases. In summary, it is a tool for automatic search of any region of the SARS-CoV-2 genome with already developed associations between disease severity, age, gender, and phylogenetic origin. These results were developed on data from GISAID, so analogously to the results we presented in our work.

In summary, the limitations of this study include the small number of prospectively tested patients. However, these results served as a reference point for the analyses using the GISAID database. The correlations between the severity of COVID-19 and the *RdRp* variability of SARS-CoV-2 was confirmed prospectively and retrospectively (using GISAID data analysis). The monitoring of the three SARS-CoV-2 SNVs mentioned above should be included in current epidemiological studies.

The results obtained clearly show that there are *loci* in the viral polymerase encoding region that can be considered mutational hot spots when it comes to the severity of symptoms caused by a particular mutant viral strain. The results also show that strains that cause severe symptoms do not have many more mutations in the polymerase encoding region. The difference between them and mildly symptom-related strains lies in the quality of the observed mutations and not in their quantity. The described single nucleotide variants may be potential targets for the development of a diagnostic test to differentiate strains that cause a high risk of hospitalization. Many publications have described new mutations correlated with the severity of COVID-19, raising alarm about the emerging variants of SARS-CoV-2. However, today these strains are not as dangerous as previously indicated by the results of this research. There is still no unequivocal evidence to clearly differentiate the virulence of SARS-CoV-2 strains. An online calculator has been developed in Poland and worldwide to assess the risk of COVID-19 severity [60, 61] but this test is based on patient-related parameters and does not consider the absolute virulence of the virus. The available routine SARS-CoV-2 diagnostic tests are mainly based on the detection of the *RdRp*, *S,* and *E* genes by real-time RT-PCR but do not detect mutations within these genes. Single commercial IVD tests distinguish, for example, SARS-CoV-2 B.1.617.2 (Delta) based on three mutations of the S gene or the B.1.1.7 variant based on the 69/70 deletion of the S gene (Vitassay Healthcare, S.L.U.). Over the last few years, since the outbreak of COVID-19, the mutations in the spike protein-encoding gene have been widely studied, which is especially important in the context of vaccinations. The general trend in research shows that mutations enhance SARS-CoV-2 in terms of infectivity and immune evasion. Given the rapid changes in this area, it is very important to pay attention to therapies that are independent of structure and functionality S - this is where research on mutations in *RdRp* comes into play [62, 63]. Despite the decline in mortality rates, COVID-19 remains the third leading cause of death in the United States [64]. The development of such tests could contribute significantly to improving the epidemiological situation worldwide and reducing the risk of infection with dangerous pathotypes.

### Supplementary Information

**Supplementary material 1.**

**Supplementary material 2.**

## Data Availability

All data generated or analyzed during this study is included in this published article and its supplementary information files (Table S1; Table S2).
